# Supplementary material for: A supernumerary synthetic chromosome in Komagataella phaffii as a repository for extraneous genetic material
Source: Microb Cell Fact. 2023 Dec 16;22:259. doi: 10.1186/s12934-023-02262-4 (PMC10724962; doi:10.1186/s12934-023-02262-4)
Supplement: Supplementary file 2 — Additional File 2 [file 12934_2023_2262_MOESM2_ESM.pdf]

A supernumerary synthetic chromosome in *Komagataella phaffii* as a repository for extraneous genetic material

Dariusz Abramczyk[1]*, María del Carmen Sánchez Olmos[2], Adán Andrés Ramírez Rojas[2], Daniel Schindler[2,3], Daniel Robertson[4], Stephen McColm[5], Adele L. Marston[6], Paul N. Barlow[1,4] *

[1] School of Chemistry, University of Edinburgh, United Kingdom
[2] Max Planck Institute for Terrestrial Microbiology, Marburg, Germany
[3] Center for Synthetic Microbiology, Philipps-Universität Marburg, Marburg, Germany
[4] School of Biological Sciences, University of Edinburgh, United Kingdom
[5] Ingenza Ltd Scotland, United Kingdom
[6] The Wellcome Centre for Cell Biology, Institute of Cell Biology, School of Biological Sciences, University of Edinburgh, United Kingdom

Additional File 1 (Supplementary figures)

Contents

Supplementary Figure S**1** *The route to our nanochromosome-carrying strains of K. phaffii*

Supplementary Figure S**2** *Confirmation of distinction between nanochromosomal CEN3 (containing additional G) and the native centromere of Chr 3*

Supplementary Figure S**3** *Preparation of framework plasmid eDA53*

Supplementary Figure S**4** *Construction of the telomeres part (Tel)*

Supplementary Figure S**5** *Validation of Tel-carrying plasmid eDA131*

Supplementary Figure S**6** *Insertion and integration arrays*

Supplementary Figure S**7** *Sequencing-coverage plots for Chrs 1-4, and the nanochromosome (nChr 1, 2A etc)*

Supplementary Figure S**8** *Genotyping by PCR is consistent with nChr 2 instability on wild-type background*

Supplementary Figure S**9** *Validating the KU70-knockout K. phaffii strain employed in this study to host nanochromosomes*

Supplementary Figure S**10** *Verification of nanochromosomes version 1 (nChr 1) and 2 (nChr 2) stability in Pichia ΔKU70 cells growing in YPD medium over 20 generations*

Supplementary Figure S**11** *Comparison of PCR-based genotyping of nChr 2A and 2B on wild-type and KU70-compromised K. phaffii backgrounds*

Supplementary Figure S**12** *Engineering a nanochromosome in the context of wild-type (CBS7435) or ΔKU70 strains of K. phaffii*

Supplementary Figure S**13** *Assessment of nChr 2A persistence in K. phaffii strain yDA260*

Supplementary Figure S**14** *Illustration of the use of an "inch-worming" strategy for in vivo gene integration into the nanochromosome that requires a ΔKU70 background*

Supplementary Figure S**15** *Validation of inch-worming*

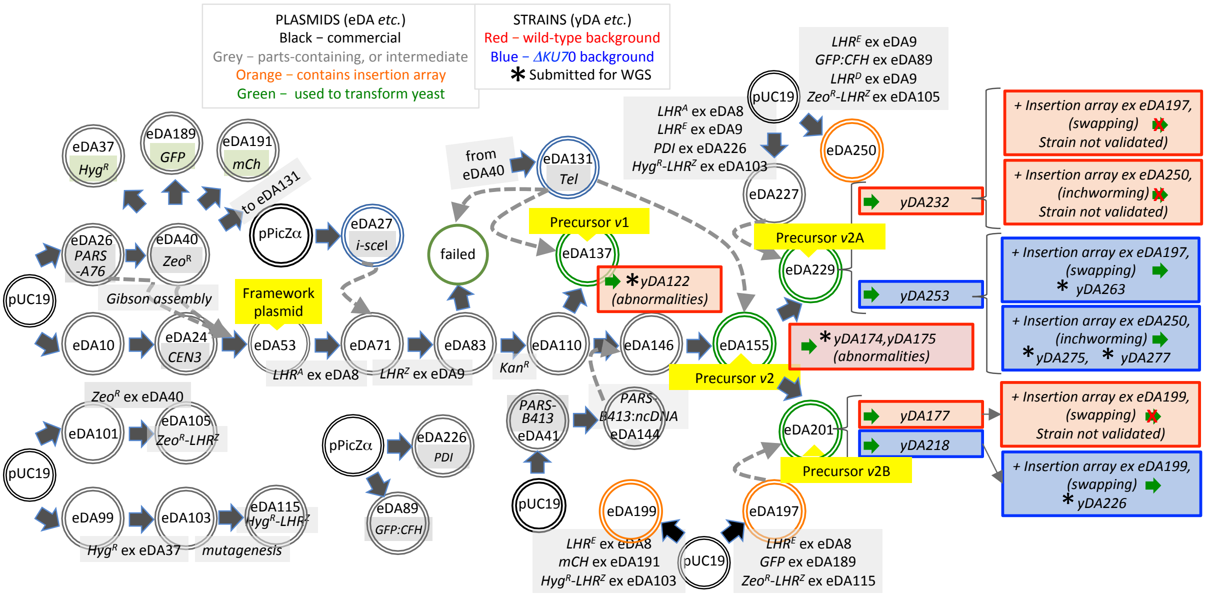

Supplementary Figure S1

The route to our nanochromosome-carrying strains of K. phaffii

This is a more detailed version of Figure 1A. Circles represent engineered-in-*E. coli* plasmids (eDAxxx etc). See also the list of plasmids in Additional file 2: Table 2 and the set of nanochromosomes drawn in Figure 1B. Boxes represents *K. phaffii* strains (yDAxxx etc) that contain nanochromosomes. See also the list of strains in Additional file 2: Table 3.

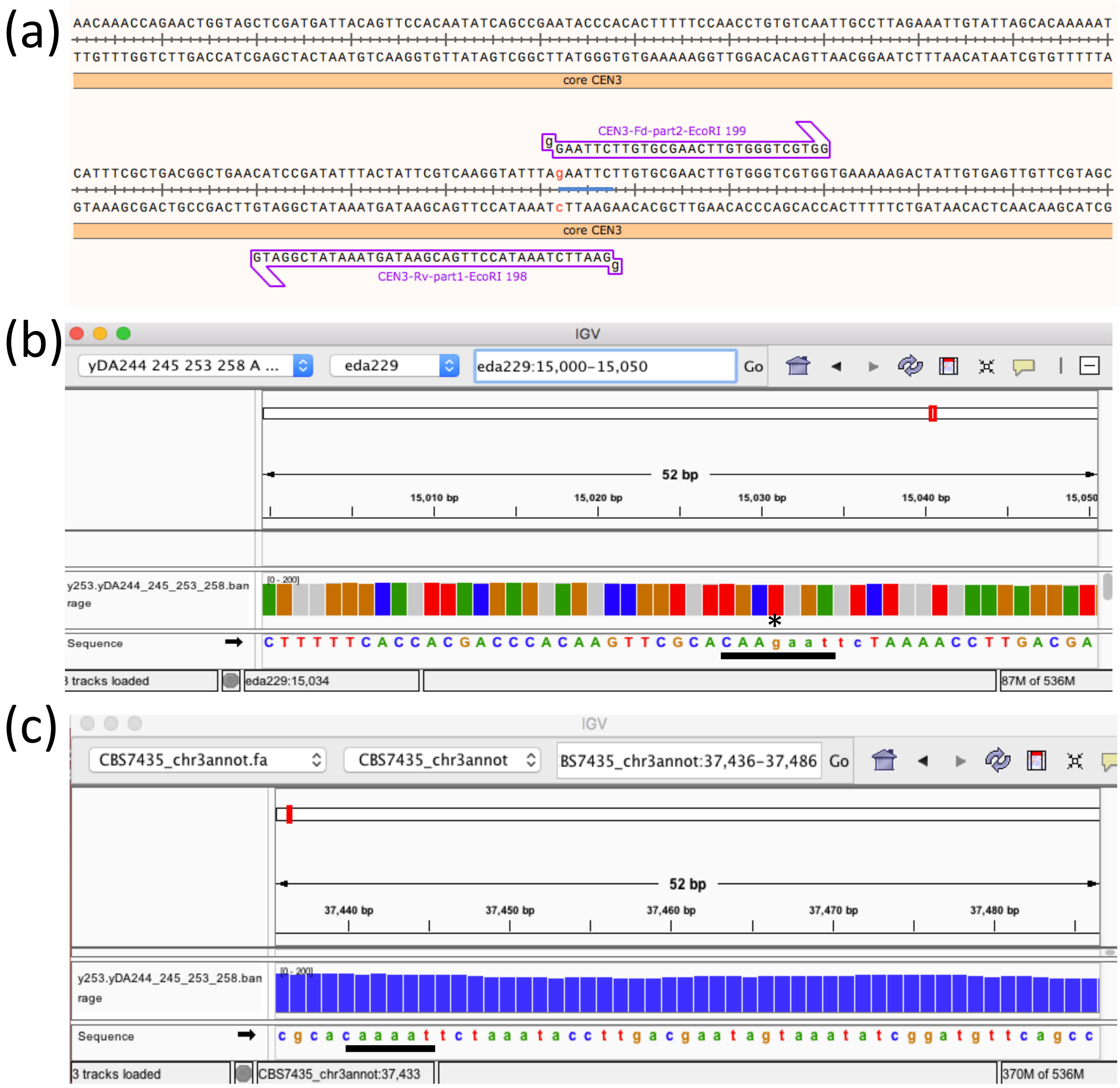

Supplementary Figure S2

Confirmation of distinction between nanochromosomal CEN3 (containing additional G) and the native centromere of Chr 3

(a) A screenshot showing SnapGene representation of core region of *CEN3* from precursor plasmid *v*2A, with a G inserted to create an *Eco*RI-recoginition site (see Fig. 2) ***(b)*** Visualization (IGV) derived from WGS of nChr 2A *CEN3* core region (from strain yDA253). The black bar shows the region containing the *inserted deoxyguanosine. ***(c)*** Visulisation (IGV) derived from WGS of the core region of the native Chr 3 centromere (from strain yDA253) . The black bar indicates the sequence that distinguishes between native (CAAAAT) and nanochromosomal (CAAGAAT) centromeres.

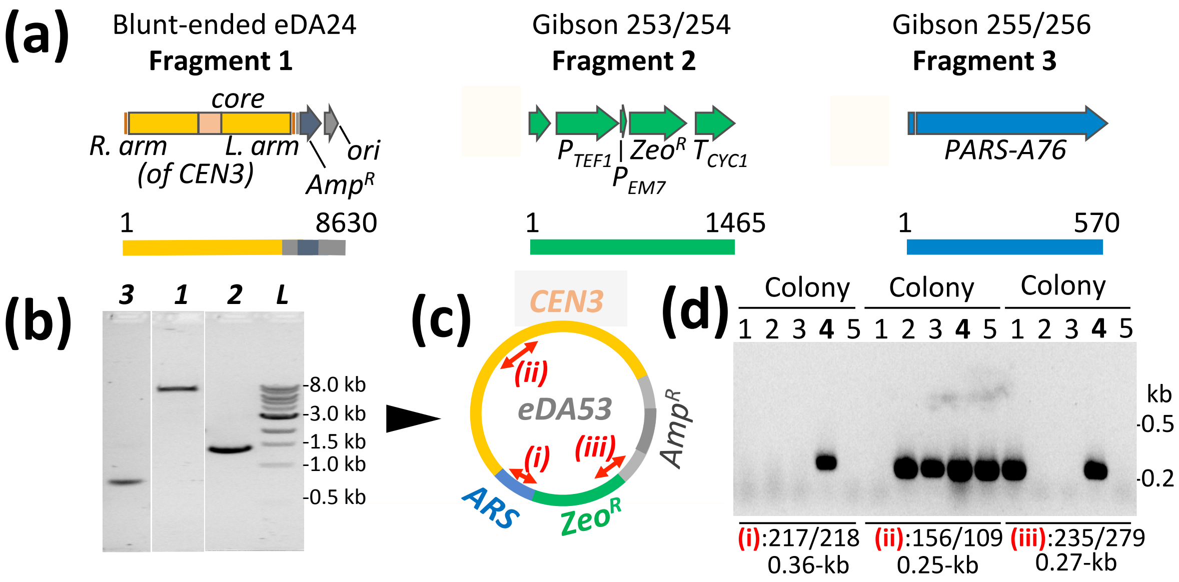

Supplementary Figure S3

Preparation of framework plasmid eDA53 (see also Fig. 2)

(a) The DNA parts (annotated by SnapGene Viewer) used for Gibson assembly. Fragment 1: eDA24 (source of *CEN3*) linearized with blunt-end restriction enzymes. Fragment 2: PCR-amplified *Zeo*^{R}, ex eDA40. Fragment 3: PCR-amplified *PARS-A76*, *ex* eDA26 (see Additional file 2: Table 1 for oligo sequences). ***(b)*** Purified fragments 1-3 ran as expected on an agarose gel. ***(c)*** Map of eDA53 with location of primer-pairs used for verification by PCR. Of five *E. coli* colonies selected on plates with 50 μg/mL zeocin and 100 μg/mL ampicillin, only one colony (number 4, highlighted) was verified, and Sanger sequencing confirmed the expected sequence of the extracted plasmid.

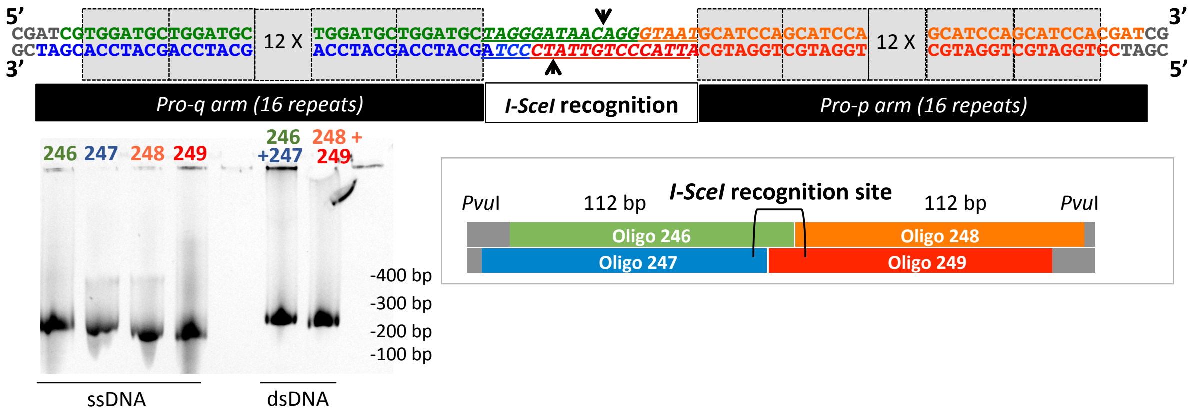

Supplementary Figure S4

Construction of the telomeres part (*Tel*) (see also Fig. 2)

The upper schematic shows the proto-telomere[*i-Sce*I-recognition site]proto-telomere structure expected after annealing and then ligation of four oligos as color-coded in the lower schematic (see Additional file 2: Table 1). Grey boxes represent some of the seven-base pair telomere repeats. The *Pvu*I-recognition site allows *Tel* excision from, and insertion into, plasmids. The confirmatory gel shows expected bands for the individual oligos (see schematic for numbering) and the products of annealing.

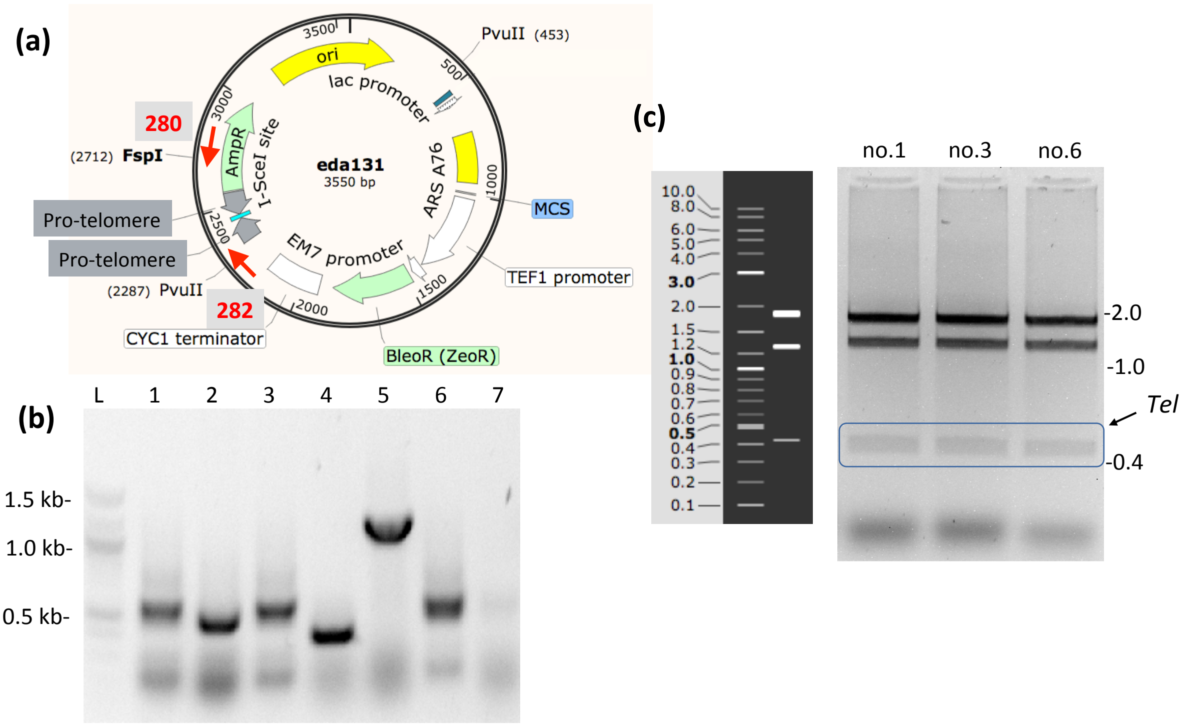

Supplementary Figure S5

Validation of Tel-carrying plasmid eDA131 (see also Fig. 2)

(a) Map of eDA131 showing the locations of the various sequences targeted by oligos (red text and arrows) used for colony PCR-based screening of *E. coli* transformants, following ligation between cleaved eDA40 and *Tel*. ***(b)*** Among zeocin-resistant and ampicillin-sensitive strains, colony-PCR using oligos 280/282 generates bands corresponding to the expected (584-bp) amplicon in clones 1, 3 and 6, only. ***(c)*** Plasmids isolated from clones 1, 3 and 6, following digestion with *Fsp*I/*Pvu*II and gel electrophoresis, yielded a pattern of bands close to the one predicted (by SnapGene). The *Tel* part could thus be digested with blunt-end restriction enzymes *Pvu*II and *Fsp*I, then gel-extracted prior to ligation with framework-plasmid eDA53.

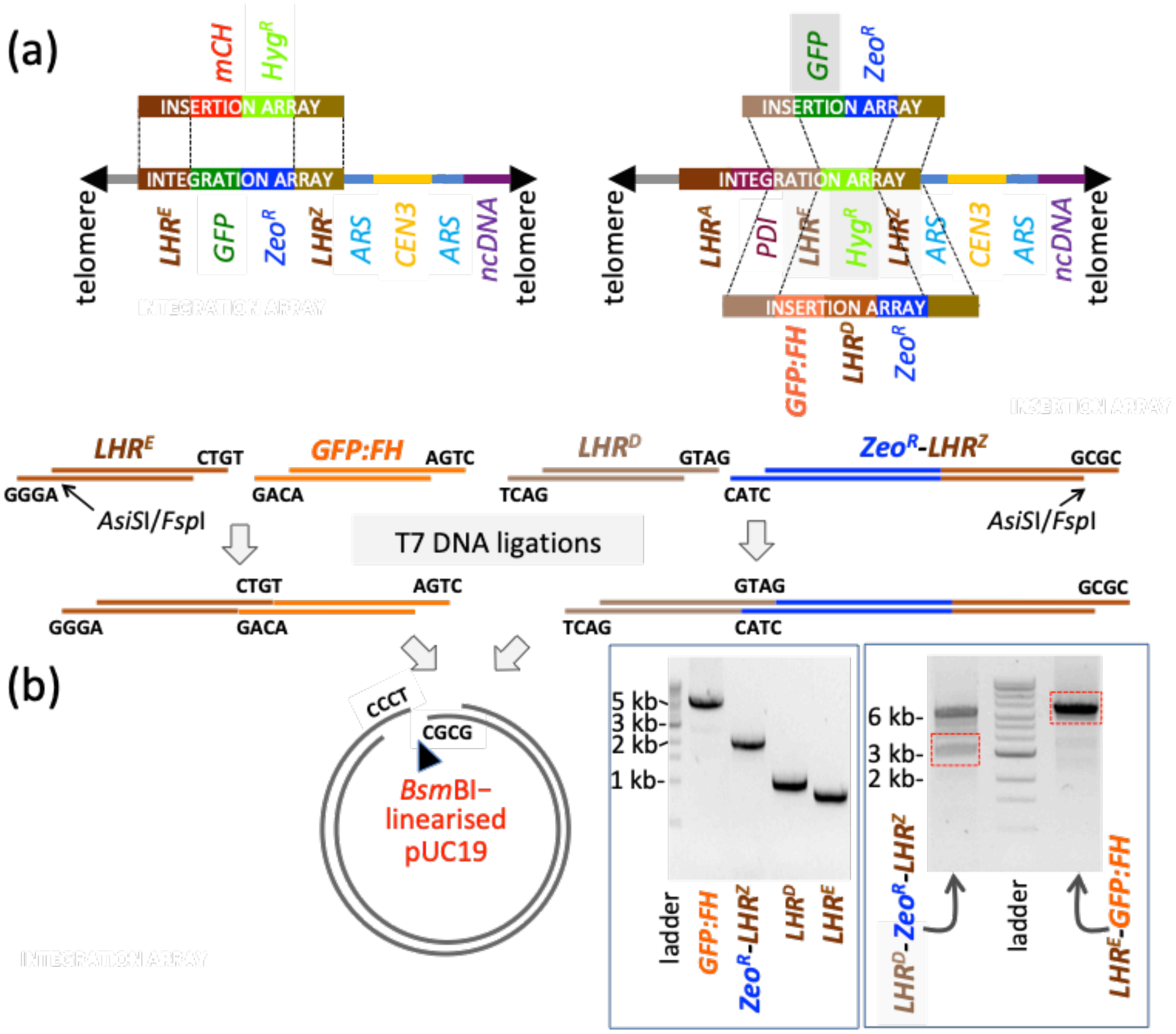

Supplementary Figure S6

Insertion and integration arrays

(a) Various strategies for integration *via* double-crossover HR of genes, delivered within an *in vitro*-assembled insertion array, into a *K. phaffii* nanochromosome-resident integration array. ***(b)*** An example of array assembly. PCR-amplified parts digested with *Bsm*BI were gel-purified then ligated pairwise. Products were gel-purified and ligated into *Bsm*BI-linearised pUC19. Resultant plasmids were used to transform *E. coli* then Sanger sequenced. In preparation for deployment, arrays were subsequently excised with *AsiS*I, or amplified by PCR (dx.doi.org/10.17504/protocols.io.bp2l69p95lqe/v1). Additional file 2: Table 2 contains a list of insertion and integration arrays.

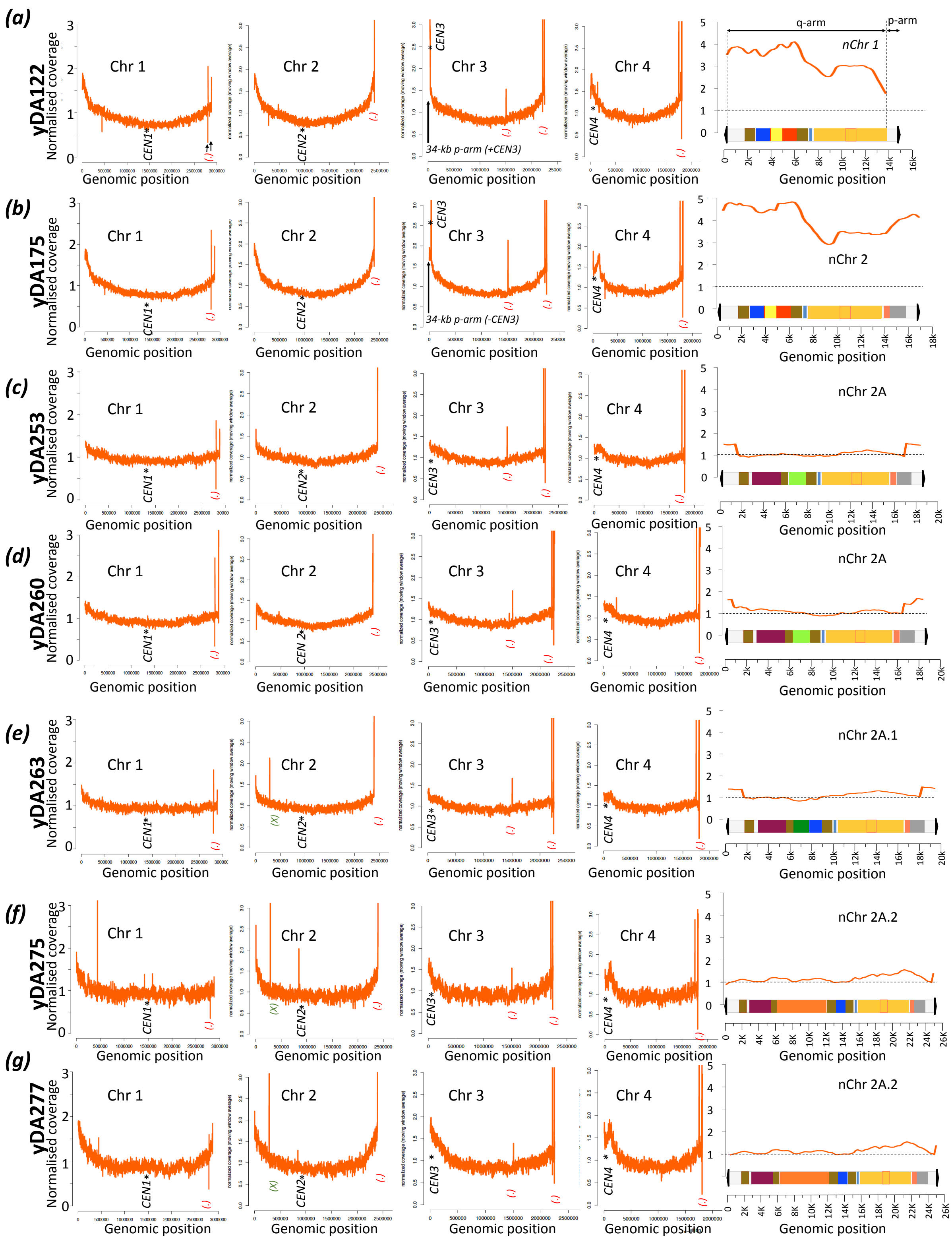

Supplementary Figure S7

Whole-genome sequencing coverage data for wild-type and ΔKU70 K. phaffii cells containing nanochromosomes (This is an extended version of Fig. 5)

See legend of Figure 5 in main text for an explanation of axes and symbols. ***(a)*** and ***(b)*:** For strains on wild-type background, plots of normalized coverage indicate two-four copies of nanochromosomal sequences per cell. Note in ***(a)*** that nChr 1 of yDA122 lacks

a p arm, while high coverage suggests there are between to and four copies, per cell, of a majority of the nChr 1 sequence. This result is compatible with *de novo* assembly (details described in the Results section, sequences deposited in Additional file 3) suggesting fusion between nChr1 q arm and the ~34-kb Chr 3 p arm. A vertical arrow in the Chr 3 plot indicates data consistent with duplication of the Chr 3 p arm and its centromere. In ***(b)***, the values (>2) for normalised coverage for nChr 2 suggest multiple copies per cell of its DNA content. The high copy-number for the Chr 3 centromere correlates with a high copy-number of nChr 2 and supports the existence of chimeric multi-centric nanochromosomes. This observation is compatible with yDA175 and yDA177 *de novo* assembly results obtained from long-read WGS (Additional file 3). ***(c)-(g):*** Strains on a Δ*KU70* background are each consistent with a single copy per cell of its nanochromosome, specifically: ***(c),*** yDA253 with a single copy of nChr 2A; ***(d),*** yDA260 with *mFH* gene integrated into (native) Chr 4 and a single copy of nChr 2A; ***(e)***, yDA263 with a single copy of nChr 2A.1 (*i.e* after replacing Hyg^{R} in nChr 2A with *GFP*-Zeo^{R}); ***(f)*** and ***(g),*** yDA275 and yDA277 (biological replicates) with single copies of nChr 2A.2 (*i.e.* after the inch-worming proof-of-principle experiment). All raw data are deposited at *BioProject PRJNA971544*.

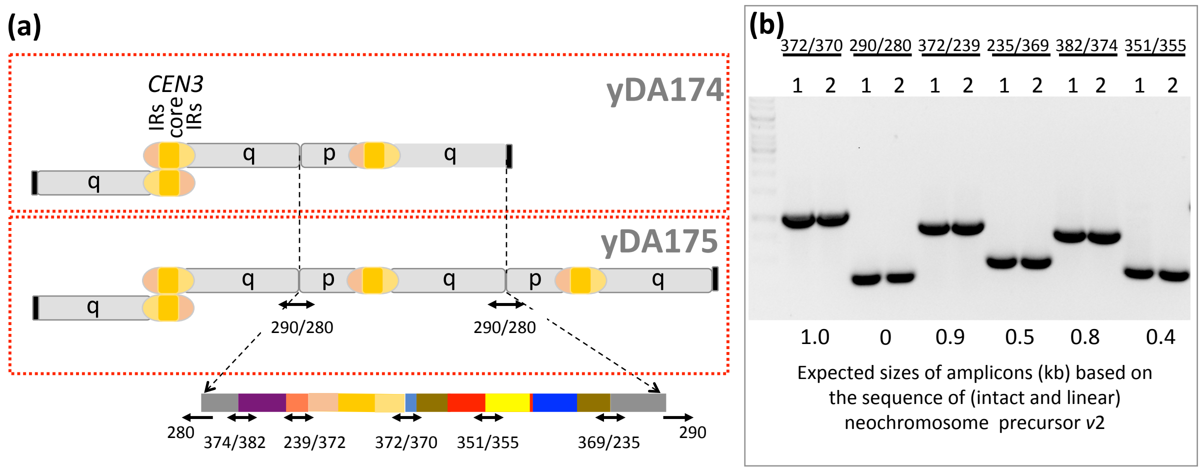

Supplementary Figure S8

Genotyping by PCR is consistent with loss of nChr 2 stability on a wild-type background (see also Fig. 4d)

(a) Schematic of multi-centric chromosomes inferred from performing *de novo* assembly analysis of the WGS of two strains of *K. phaffii* created by transforming wild-type cells with nChr 2, followed by cell culture for multiple generations. A color-coded (not to scale, see Fig. 1B) map of nChr 2 (telomeres not shown) is drawn below with the sites targeted by oligo pairs (see Additional file 2: Table 1) for PCR-based genotyping. ***(b)*** Agarose gel showing PCR amplicons derived from the oligo pairs shown in panel (a); (1) for yDA174, (2) for yDA175. The oligo pair 290/280 (lanes 3 and 4 on the gel) yields a ~0.4 kb amplicon, consistent with chromosome fusion. This would not have been detected had nChr 2 remained intact.

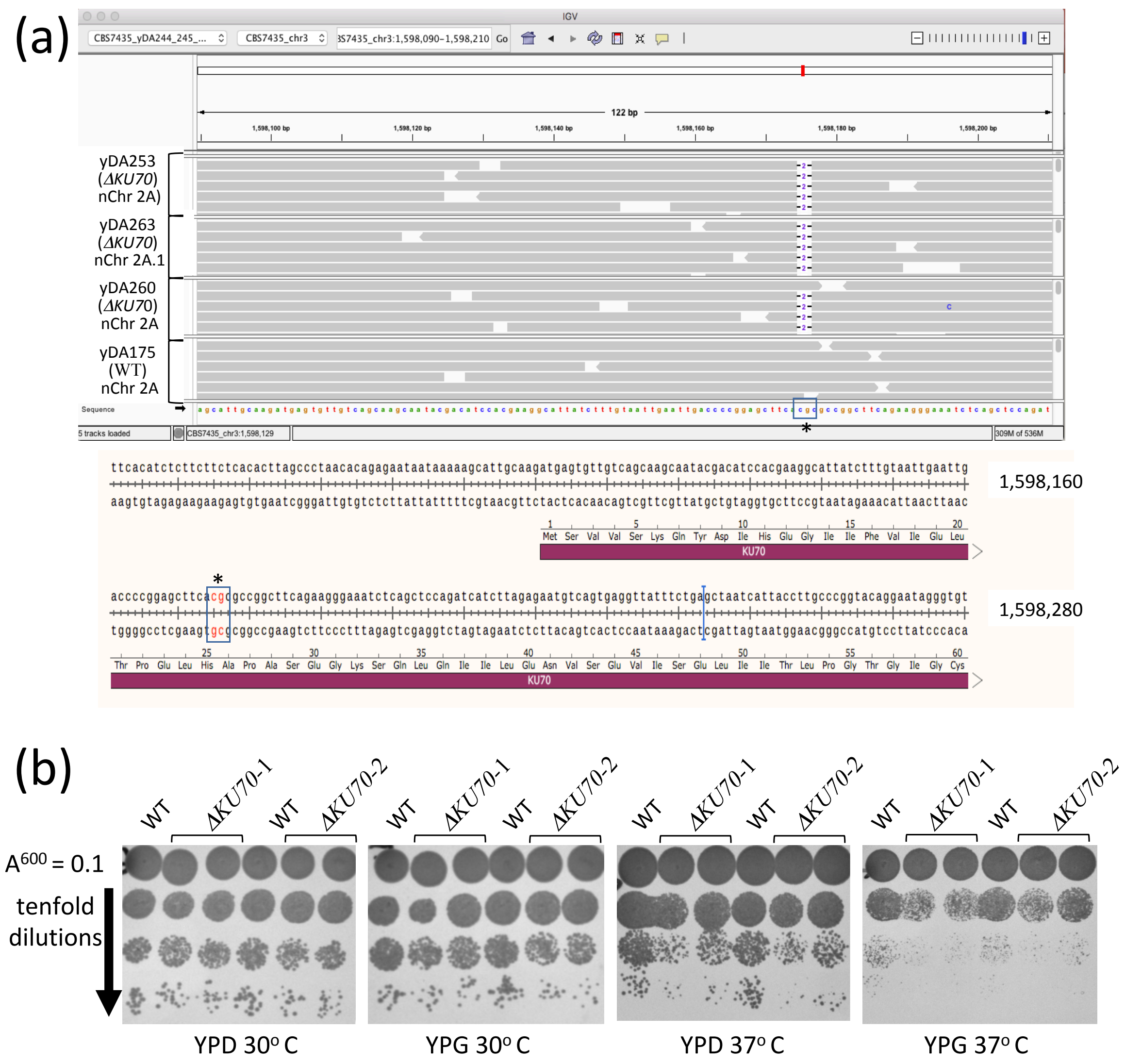

Supplementary Figure S9

Validating the KU70-knockout K. phaffii strain employed in this study to host nanochromosomes

(a) Upper box: Annotated visualisation (IGV) of WGS results confirms deletion of CG dinucleotide spanning codons for *KU70* ^{25}His-Ala^{26}. The *KU70* start codon is indicated with a black rectangle. Lower box: Annotated SnapGene screenshot showing the sequence *of KU70* (on Chr 3) and highlighting the dinucleotide (GC) deleted in our *ΔKU70* strain. ***(b)*** Serial dilution to compare viabilities of wild-type and *ΔKU70* strains under various conditions. CBS7435 (WT) cells and two *ΔKU70* isolates, yDA208 (*ΔKU70*-1) and yDA210 (*ΔKU70*-2) were inoculated into YPD and grown for 24 hours at 30 °C. Cultures were adjusted to A^{600} = 0.1, then subjected to a series of tenfold dilutions for plating onto YPD or YPG (glycerol) agar. Plates were incubated at 30 °C for two days, or 37 °C for four days.

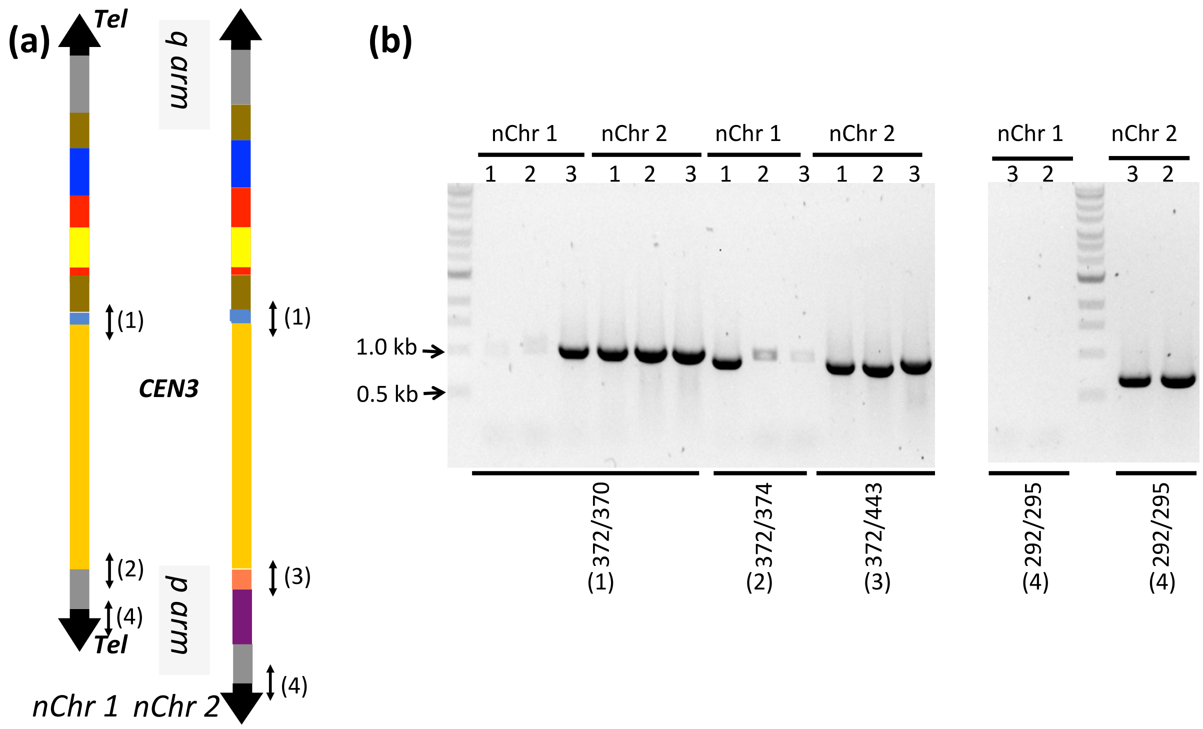

Supplementary Figure S10

Verification of nChr 1 and nChr 2 stability in ΔKU70 cells growing in YPD medium for 20 generations

PCR-based genotyping confirms integrity of extended version (nChr 2) in contrast to shorter (nChr 1) version for which one or more crucial bands are absent from each strain. ***(a)*** Primers were designed to target regions linking *CEN3* and the q arm of both nanochromosomes (370/372; primers set 1) or the p arm of either nChr 1 (372/374; set 2) or nChr 2 (372/443; primers set 3). Primer set 4 (292/295) validates the p-arm telomere of both nanochromosomes. ***(b)*** Note the absence of an amplicon corresponding to the p arm telomere of nChr 1, in contrast to nChr 2. An amplicon for set 2 is missing or weak in two out of three colonies transformed with nChr 1.

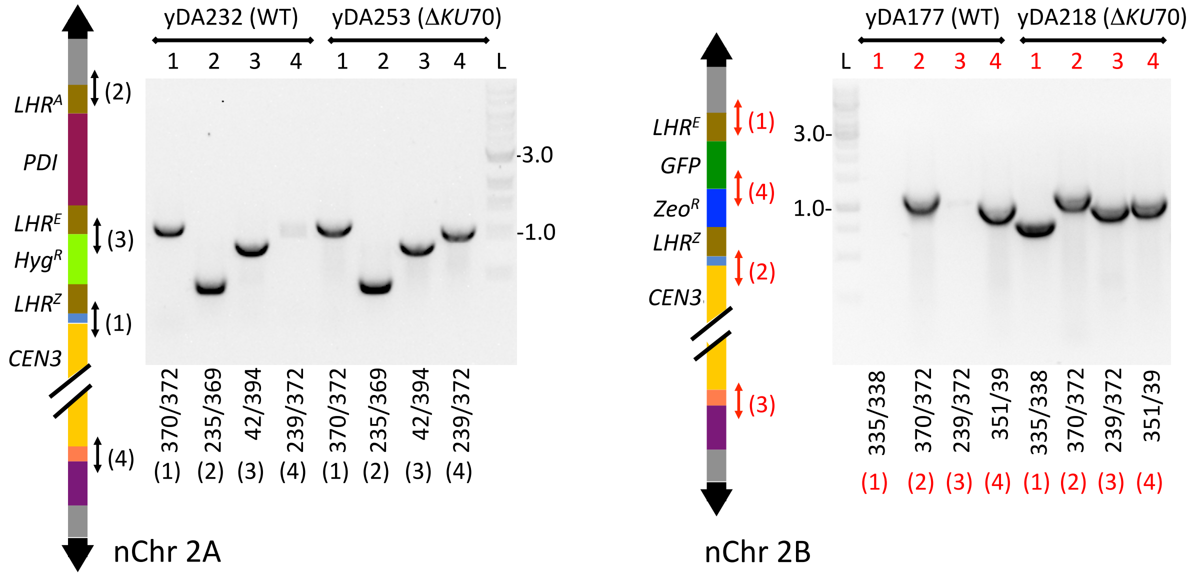

Supplementary Figure S11

Comparison of PCR-based genotyping of nChr 2A and nChr 2B on wild-type and ΔKU70 K. phaffii backgrounds

Missing amplicons in the cases of yDA232 (oligo pair 4) and yDA177 (oligo pairs 1 and 3) are consistent with the instability of both these extended nanochromosomes on wild-type backgrounds. Conversely, candidate bands are evident for all four targets in both the *ΔKU70* strains, *i.e.* yDA253 and yDA218. Analysis was performed on strains inoculated and cultivated on YPD medium, without antibiotic, for 24 hours. These data are in good agreement with WGS.

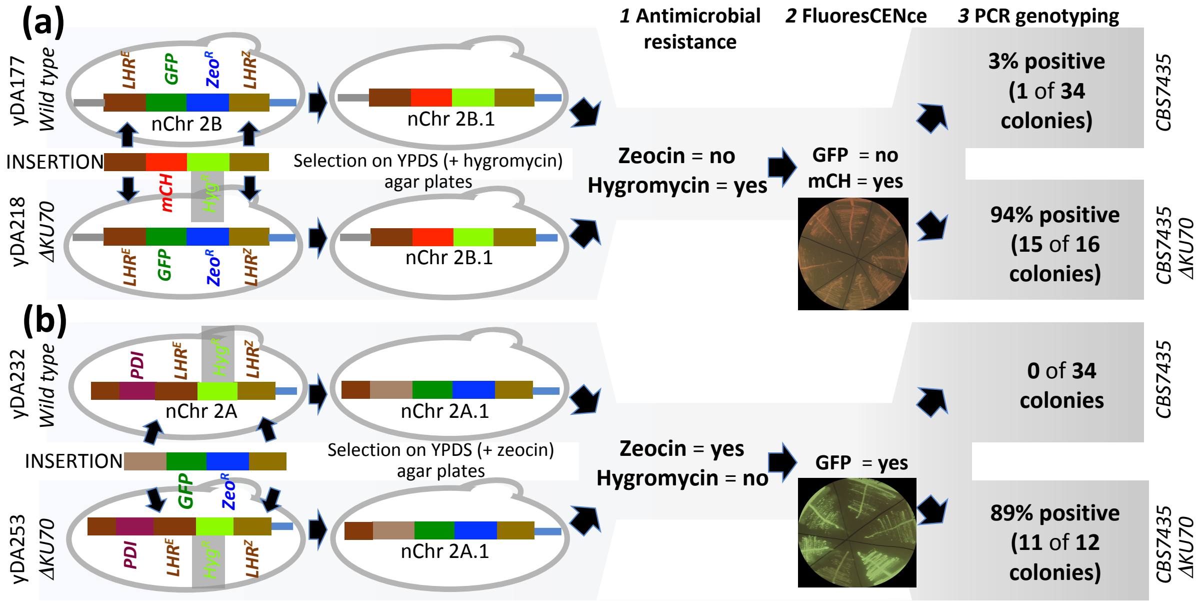

Supplementary Figure S12

Engineering a nanochromosome in the context of wild-type (CBS7435) or ΔKU70 strains of K. phaffii (See also Figure 7)

These proof-of-principle experiments were designed to assess the feasibility of replacing genes within the landing zones of nanochromosomes with different ones, *in vivo*, by double cross-over HR. ***(a)*** Attempted replacement of *GFP-Zeo*^{R} in the landing zone of nChr 2B with *mCH-Hyg*^{R}. ***(b)*** Attempted replacement of *Hyg*^{R} in the landing zone of nChr 2A with *GFP* and *Zeo*^{R}. Note the higher success rate obtained with *ΔKU70* strains in terms of selected colonies.

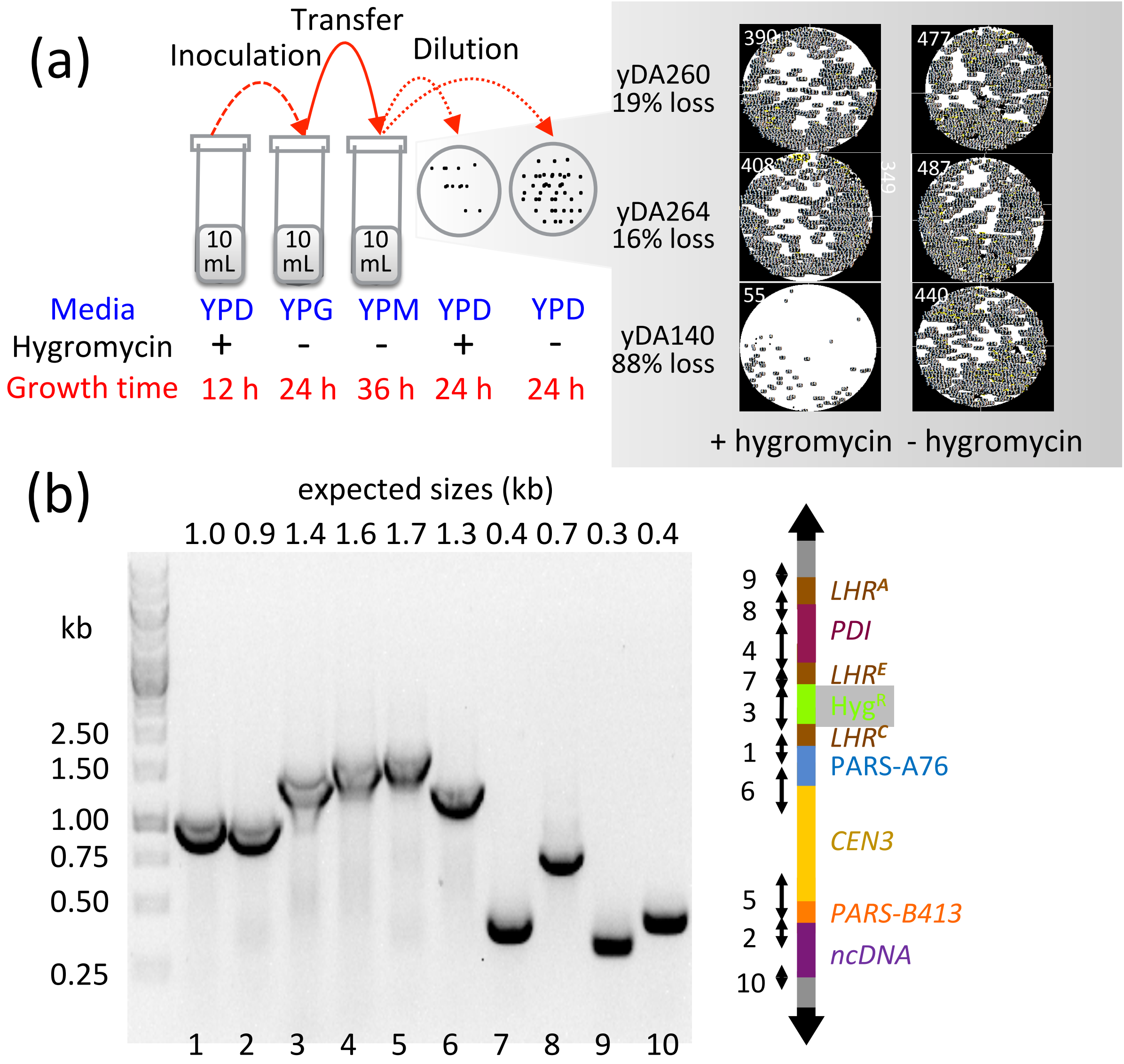

Supplementary Figure S13

Assessment of nChr 2A persistence in K. phaffii strain yDA260

(a) Cells grown for 12 hours in YPD (+ hygromycin) were used to inoculate YPG (no antibiotic). After 24 hours of exponential growth in YPG, cells were spun down then re-suspended in YPM (no antibiotic). After 36 hours of methanol-induction, calls were streaked out on YPD plates, with or without hygromycin. An assessment of the retention of *Hyg*^{R} (and, by extension, nChr 2A) was made by comparing colony counts with *versus* without hygromycin. The results (right-hand panels, white numerals are colony counts derived from ImageJ software) were expressed as % of colonies that were no longer-hygromycin resistant (note: yDA260 carries nChr 2A; yDA264 is a control in which the *Hyg*^{R} cassette is integrated into the native genome; yDA140, contains only (telomere-null/centromere-null) eDA37 (see Additional file 2: Table 5). ***(b)*** Representative genotyping by colony PCR *of K. phaffii* strain yDA260 recovered from YPD plate after the chromosome-loss assay. The (numbered) sites targeted by oligo-pairs (Additional File 2: Suppl. Table 2) are indicated on on the schematic of nChr 2A.

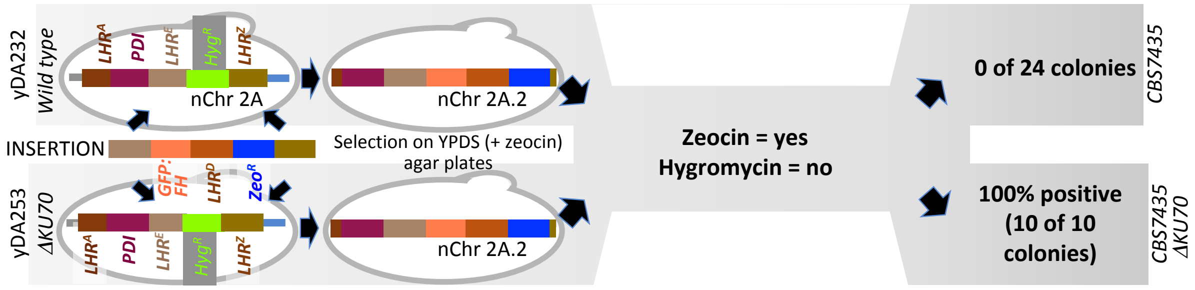

Supplementary Figure S14

Illustration of the use of an "inch-worming" strategy for in vivo gene integration into the nanochromosome that requires a ΔKU70 background.

The wild-type and *ΔKU70* strains shown were transformed with an integration array, schematized in the figure, which was designed to replace *Hyg*^{R} in nChr 2A with *GFP:FH-LHR*^{D}*-Zeo*^{R}. Single colonies that grew on zeocin-containing agar plates were screened for zeocin resistance and hygromycin sensitivity followed by PCR genotyping. Only *KU70*-compromised strains tested positive.

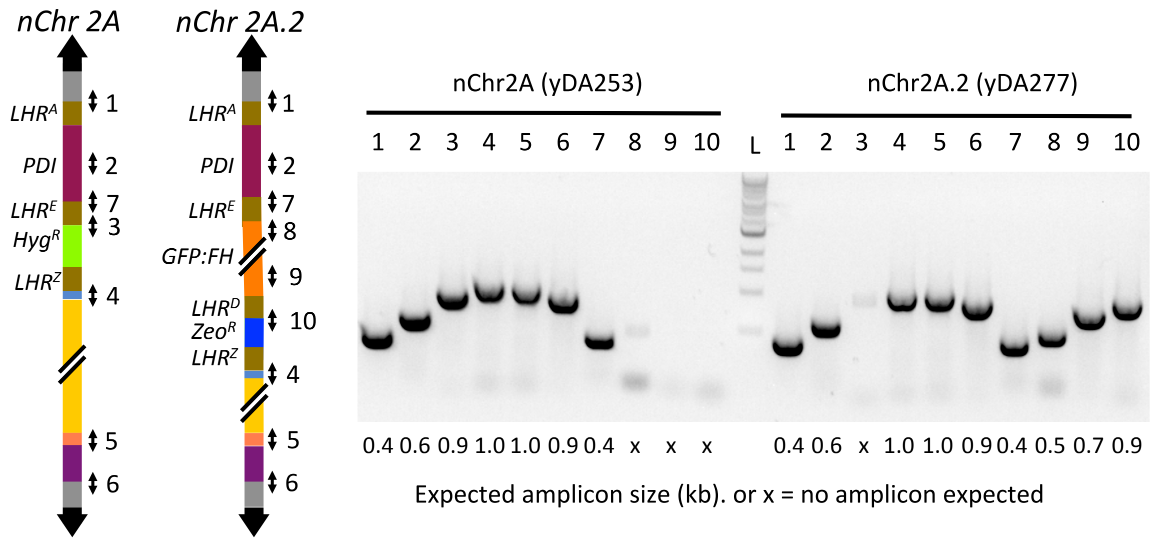

Supplementary Figure S15

Validation of inch-worming

Here, nChr 2A was engineered, *in vivo*, into nChr 2A.2, by gene integration accompanied by simultaneous extension of the landing zone (ready for a future integration) and by exchange of selection-markers. *ΔKU70 K. phaffii* cells carrying nChr 2A were transformed with the insertion array *LHR^{E}-GFP*:*FH-LHR^{D}-Zeo^{R}-LHR^{Z}*. Transformants (growing on zeocin) were transferred to YPD agar plates, then screened for zeocin resistance and hygromycin sensitivity. An attempt was made to validate nChr2A.2 by PCR-based genotype mapping. The amplicon numbers shown in the carton arise from the following ten oligo pairs (see Additional file 2: Table 1): (1) 369/235; (2) 23/25; (3) 394/372; (4) 370/372; (5) 443/372; (6) 382/374; (7) (394/338); (8) 394/272; (9) 274/278; (10) 40/345.
