# Supplementary material for: A supernumerary synthetic chromosome in Komagataella phaffii as a repository for extraneous genetic material
Source: Microb Cell Fact. 2023 Dec 16;22:259. doi: 10.1186/s12934-023-02262-4 (PMC10724962; doi:10.1186/s12934-023-02262-4)
Supplement: Supplementary file 3 — Additional File 3 [file 12934_2023_2262_MOESM3_ESM.pdf]

A supernumerary synthetic chromosome in *Komagataella phaffii* as a repository for extraneous genetic material

Dariusz Abramczyk[1], María del Carmen Sánchez Olmos[2], Adán Andrés Ramírez Rojas[2], Daniel Schindler[2,3], Daniel Robertson[4], Stephen McColm[5], Adele L. Marston[6], Paul N. Barlow[1,4]

[1] School of Chemistry, University of Edinburgh, United Kingdom
[2] Max Planck Institute for Terrestrial Microbiology, Marburg, Germany
[3] Center for Synthetic Microbiology, Philipps-Universität Marburg, Marburg, Germany
[4] School of Biological Sciences, University of Edinburgh, United Kingdom
[5] Ingenza Ltd Scotland, United Kingdom
[6] The Wellcome Centre for Cell Biology, Institute of Cell Biology, School of Biological Sciences, University of Edinburgh, United Kingdom

Additional file 2 (Tables)

Contents

Table 1 *List of oligonucleotides used in this study*
Table 2 *List of plasmids used in this study*
Table 3 *List of K. phaffii strains referred to in the current work*
Table 4 *List of insertion and integration arrays*
Table 5 *Results of (nano)chromosome-loss assays.*
Table 6 *Estimation of gene-copy number*

Table 1 *List of oligonucleotides used in this study*

**Oligo ID number (F = forward; R = reverse)**	**Purpose/description**	**Sequences (5'- 3')** Type-II restriction enzyme-recognition sites underlined; **type-IIS restriction enzyme-recognition sites in bold**; lower case letters denote overhangs generated by type-IIS restriction enzymes
23 (F)	Recombinant *PDI*	GCCAGAGGACGCTTCTAACTTGG
25 (R)	Recombinant *PDI*	GCTGGAGTGGAGTCGGAAGTG
42 (R)	For genotyping (*hph*)	GGCGCAGCTATTTACCCGC
132a (R)	T_{CYC1} *(BsmBI)*	G**CGTCTC**CgtagGCCTTCGAGCGTCCC
156 (F)(R)	Internal Repeats of *CEN3.* For genotyping (Fig. 4C, in set 6)	CAGGCTACCTCATATCTCCATCATC
197 (F)	*CEN3* part (*Kpn*I)	GCGGTACCGTAGATCAAGTGACTTCTTGAGCTTGCC
198 (R)	*CEN3* part (*Eco*RI)**;** an additional **G** is inserted into nanochromosomal *CEN3* cores (Fig. 4C, in set 6)	G**G**AATTCTAAATACCTTGACGAATAGTAAATATCGGATG
199 (F)	As above	G**G**AATTCTTGTGCGAACTTGTGGGTCGTGG
200 (R)	*CEN3* part (*Nde*I)	CCTTCCCCATATGTGCTCCGTCAGCTTGAATAAGCC
212 (R)	Murine *FH* (for qPCR)	CCGTCGCCATTCTCGACTC
215 (F)	Murine *FH* (for qPCR)	GCAGCCGAAACCGATCAGGAG
217 (F)	*PARS-A76 (BamH*I*)*	GGGGATCCGGCGCTCCAACTGTATTCTAGG
218 (R)	*PARS-A76 (BamH*I*)*	GGGGATCCCGAAGAGATGAGATGAGCTGATAAGATG
223 (F)	*iSce*I*-ORF (EcoR*I*)*	GGAATTCATGGGATCAAGATCGCCAAAAAAGAAG
224 (R)	*iSce*I*-ORF (Sac*II*)*	GCCGCGGTTATTTCAGGAAAGTTTCGGAGGAGATAGTG
235 (F)	pUC region (Fig. 4C, in set 1)	AGCGAGTCAGTGAGCGAG

238 (F)	*PARS-B413* (*BamH*I)	GCGGATCCCAACAATACTACGGAAACGACCCA
239 (R)	*PARS-B413* (*BamH*I) (Fig. 4C, in set 5)	GGGGATCCGATAAGCTGGGGGAACATTCGC
246 (F)	5'-*Tel*, complementary strand to 247 (*in italic*s – *I-Sce*I-recognition site part) – includes *Pvu*I	CGTGGATGCTGGATGCTGGATGCTGGATGCTGGATGCTGGATGCTGGATGCTGGATGCTGGATGC TGGATGCTGGATGCTGGATGCTGGATGCTGGATGCTGGATGCTGGATGC*TAGGGATAACAGG*
247 (R)	Complementary to strand 246 (*in italic*s – *I-Sce*I-recognition site part) – includes *Pvu*I	*CCTA*GCATCCAGCATCCAGCATCCAGCATCCAGCATCCAGCATCCAGCATCCAGCATCCAGCATCCA GCATCCAGCATCCAGCATCCAGCATCCAGCATCCAGCATCCAGCATCCACGAT
248 (F)	3'-*Tel*, complementary strand to 249 (*in italics* – *I-Sce*I-recognition site part) – includes *Pvu*I	*GTAAT*GCATCCAGCATCCAGCATCCAGCATCCAGCATCCAGCATCCAGCATCCAGCATCCAGCATC CAGCATCCAGCATCCAGCATCCAGCATCCAGCATCCAGCATCCAGCATCCACGAT
249 (R)	3'-*Te*l, complementary strand to 248 (*in italics* – *I-Sce*I-recognition site part) – includes *Pvu*I	CGTGGATGCTGGATGCTGGATGCTGGATGCTGGATGCTGGATGCTGGATGCTGGATGCTGGATGC TGGATGCTGGATGCTGGATGCTGGATGCTGGATGCTGGATGCTGGATGC*ATTACCCTGTTATC*
253 (R)	1.4-kb Gibson assembly fragment 1 for framework plasmid	GAAATTGTTATCCGCTCACAATTCGCGGCCTTTTTACGGTTCCT
254 (F)	1.4-kb Gibson assembly fragment 1 for framework plasmid	CCCGCGCGTTGGCCGATTCATTAATGCAGTTGTCATTTTGTTTCTTCTCGTACGAGCTTG
255 (F)	0.51-kb Gibson assembly fragment 2 for framework plasmid	AGGAACCGTAAAAAGGCCGCGAATTGTGAGCGGATAACAATTTCACACAG
256 (R)	0.51-kb Gibson assembly fragment 2 for framework plasmid	TGCTTGGCAAGCTCAAGAAGTCACTTGATCTACGGTACCCGGGATGTGCTGCAAGGCG
264 (F)	*LHR*^{A} (*Pvu*II)	AACAGCTGTGTTAAACCGTCTTTAAG
267 (F)	*i-Sce*I cassette part (*BsmB*I)	C**CGTCTCA**gggaATCTAACATCCAAAGACGAAAGGTTG
268 (R)	*i-Sce*I cassette part (*BsmB*I)	C**CGTCTCA**ttggCTCACTTAATCTTCTGTACTCTGAAGAGG
269 (F)	*LHR*^{Z} part (*BsmB*I)	C**CGTCTCC**ccaaCCGTGTAGGTCTACAAACTGG

270 (R)	*LHR*^{Z} part (*BsmB*I)	C**CGTCTCA**cgcgCCTGCAGGTTGAGTTGGCGAAGGTGCG
272 (R)	P_{AOX1} (for genotyping)	CCTCCCCTATGTGATCGTCG
273 (R)	*LHR*^{A} (*Pvu*II)	AACAGCTGCAAACTAAGATTGGACTATTGCTATCC
274 (F)	*GFP* (for genotyping)	AAGTTGCCCGTCCCCTG
276 (F)	*GFP*-linker-fusion (*Nsi*I)	CCCATGCATCCATGGTATCTAAAGGTGAAGAACTATTCACAG
277 (R)	*GFP*-linker-fusion (*Nsi*I)	CCCATGCATGACGACCTTCAATTTTGTAAAGCTCATCCATTCCCAAG
278 (R)	*Human FH* (for genotyping)	CCCACTCACCCTTTCTACAAACC
280 (R)	*Amp*^{R} (Fig. 4C, in set 2)	TTATCCGCCTCCATCCAG
282 (R)	*M13* (genotyping)	CTGGCCGTCGTTTTACAACG
290 (F)	*Amp*^{R} (Fig. 4C, in set 2)	CTATGTGGCGCGGTATTATC
300 (F)	*LHR*^{A} *(BsmB*I*)*	C**CGTCTCC**gggaCTGTGTTAAACCGTCTTTAAGTCAAC
301 (R)	*LHR*^{A} *(BsmB*I*)*	G**CGTCTCC**TgtccAAACTAAGATTGGACTATTGCTATC
302 (F)	P_{AOX1} (*BsmB*I)	C**CGTCTC**CgacaAACATCCAAAGACGAAAGGTTG
302a (F)	P_{TEF1} (for *GFP* and *mCH* cassettes) (*BsmB*I)	C**CGTCTC**CgacaGTTTCTACTCCTTTTTTACTCTTCC
303 (R)	T_{AOX1} (*BsmB*I)	G**CGTCTC**CctgaTCTCACTTAATCTTCTGTACTCTG
303a (R)	T_{CYC1} (for *GFP* and *mCH* cassettes) (*BsmB*I)	G**CGTCTC**CctgaGCCTTCGAGCGTCCC
304 (F)	*LHR*^{D} (*BsmB*I)	C**CGTCTC**CtcagGACAGCAACCTAACCGAC
305 (R)	*LHR*^{D} (*BsmB*I)	G**CGTCTC**CctacCAGTCCTCGTGAAAGACGAG
306 (F)	*Hyg*^{R} (*Sal*I-*Bsa*I)	CCTGTCGAC**GGTCTC**AgtagGACATGGAGGCCCAGAATACCC
307 (R)	*Hyg*^{R} (*BamH*I)	GCGGATCCCAGTATAGCGACCAGCATTCACATAC
308 (F)	*Zeo*^{R} cassette (*Sal*I-*BsmB*I) overhang	CTGTCGAC**CGTCTC**AgtagCCCACACACCATAGCTTCAAAATG

	GTAG	
309 (R)	Zeo^R cassette (*BamH*I)	GGGATCCGCAAATTAAAGCCTTCGAGCG
315 (F)	LHR^A *(BsmB*I-*AsiS*I*)*	CCGTCTCCgggaGCGATCGCCTGTGTTAAACCGTCTTTAAGTCAACCC
317 (R)	LHR^Z (*Kpn*I-*Bsa*I-*AsiS*I)	GGGGTACC**GGTCTC**AcgcgGCGATCGCCCTGCAGGTTGAGTTGGCGAAGGTGCG
318 (R)	LHR^Z (*Kpn*I-*BsmB*I-*AsiS*I)	GCGGTACC**CGTCTC**AcgcgGCGATCGCCCTGCAGGTTGAGTTGGCGAAGGTGCG
321 (F)	Bacterial Kan^R cassette (*Sph*I)	CGCGCATGCTGATCGGCACGTAAGAGG
322 (R)	Bacterial Kan^R cassette (*Sph*I)	GCGGCATGCCGCAGAAAGGCCCACCCG
335 (F)	LHR^E (*Bsa*I-*AsiS*I)	C**GGTCTC**CgggaGCGATCGCGACTTACCTCGTTTTAACTTAGTCGG
336 (R)	LHR^E (*Bsa*I)	C**GGTCTC**CTtcagGACTTACCTCGTTTTAACTTAGTCGG
338 (R)	LHR^E (*Bsa*I)	G**GGTCTCC**tgtcACAAGTTTAACTAAGCGTCAGCC
345 (F)	LHR^D (genotyping)	CGGCAATCCTGTACTCTGC
351 (R)	LHR^Z verification (Fig. 4C, in set 3)	GCTTCTGCTCTTAAACTAGCTGC
355 (F)	T_{AOX1} (Fig. 4C, in set 3)	TCGTACGAGCTTGCTCCTG
369 (R)	LHR^A	CTGTTGAGGCACAAACCGTCAG
370 (F)	LHR^C (qPCR, genotyping) (Fig. 4C, in set 4)	CGTTACACGGCAGTCCTGTTCC
372 (F)(R)	Internal repeats /CEN*3* (genotyping) (Fig. 4C, in sets 4 and 5)	CCGTTGGACCAATGTGGAATC
374 (R)	3'-p arm (genotyping)	ATAATACCGCGCCACATAGC
375 (R)	3'-p arm of Chr 3 (genotyping)	CATGTGTATTGGAGAGCAGAG
379 (R)	1.3-kb ncDNA (*Nde*I)	CCTTCCCCATATGCTAGTGCAGAGTGAGCCTG
380 (F)	1.3-kb ncDNA (*EcoR*I)	GGAATTCGCTAGCCAGATCGGCTTAAG
381 (F)	1.9-kb *ncDNA-PARS-B413* (*Nde*I)	CCTTCCCCATATGCAACAATACTACGGAAACGACCC

382 (F)	1.3-kb *ncDNA* verification (Fig. 4C, in set 7)	GATTCGCCCTGCAGATTC
384 (F)	Amplification of insertion array (*Afe*I), blunt end (*LHR*^{A}-)	CCCAGCGCTGTGTTAAACCGTCTTTAAG
394 (F)	*LHR*^{E} verification	GCCAACCAAAGGTGGAGG
397 (F)	Amplification of insertion array (*Afe*I) blunt end (*LHR*^{E}-)	TTATAGCGCTGACTTACCTCGTTTTAACTTAGTCGG
409 (R)	Amplification of insertion array (*LHR*^{Z}-) (*XhoI*) (*SalI*I compatible end)	TTATCTCGAGTGAGTTGGCGAAGGTGCG
417 (R)	*LHR*^{Z} verification	TTGAGTTGGCGAAGGTGCG
443 (R)	Verification of 1.9-kb *ncDNA-PARS-B413*	AAAGTCAACTGTCCTTTCTGGC
448 (R)	*PDI* part (*Not*I) introducing HIS-tag-TAA stop codon (*italics*)	TTTTTGCGGCCGC*TTAATGATGATGGTGGTGATG*CAACTCATCGTGAGCATCAGCTTC

Table 2 *List of plasmids used in this study*

Plasmid ID	Size (Kb)	Key contents (notes)	Purpose	Source
pUC19	2.68		Cloning	Thermo Fisher Scientific
pPICZα B	3.6		Cloning and expression	Thermo Fisher Scientific
pPICZ A	3.3		Cloning and expression	Thermo Fisher Scientific
pGS-GnT-II	5.8		Cloning	Glycoswitch
pGS-GnT-I	4.2		Cloning	Glycoswitch
eDA8	10.5	Contains 8 kb of DNA designed to be non-coding, have no restriction sites *etc.*	Source of *LHRs*$^{A\text{-}D}$, and *ncDNA* extension in p-arm of nanochromosome	In house (V. Zulkower & A. Marston, unpublished)
eDA9	10.5	Contains 8 kb of DNA designed to be non-coding, have no restriction sites *etc.*	Source of *LHR*^{E}, *LHR*^{D}, *LHR*^{Z}	In house (V. Zulkower & A. Marston, unpublished)
eDA10	5.92	Chr 3 centromere (*CEN3)* (5'-part) in pUC19	En route to eDA24	This work
eDA22	6.98	pRS413-*(*P_{GAL}*)I-SceI*	Source of meganuclease gene	(Shen, Wang et al. 2017)
eDA24	8.85	*CEN3* in pUC19	DNA-parts assembly (centromere)	This work
eDA26	3.0	*pARS-A76* in pUC19	DNA-parts assembly (ARS)	This work
eDA27	4.12	pPICZ A-(P_{AOX1})*I-SceI(*T_{AOX1}*)*	Meganuclease gene cassette, intended for *in vivo* linearisation	This work
eDA37	4.2	*PARS-A76-Hyg*^{R} in pUC19	Source of *Hyg*^{R} and negative control for chromosome-loss assay	This work
eDA40	4.2	*PARS-A76-Zeo*^{R} in pUC19	Source of *Zeo*^{R} and negative control for chromosome-loss assay	This work
eDA41	3.2	*PARS-B413* in pUC19	DNA-parts preparation (ARS)	This work
eDA53 (framework	10.5	pUC-*PARS-A76-CEN3-Zeo*^{R}	Framework plasmid (see Fig. 1A)	This work

plasmid)				
eDA71	11.3	pUC-*PARS-A76-CEN3-LHR*^{A}	Intermediate plasmid en route to eDA83	This work
eDA83	14.5	pUC-*PARS-A76/CEN3-LHR*^{A}- *Zeo*^{R}-*(P*$_{AOX1}$*)I-SceI-LHR*^{Z}	Abandoned strategy for self-cleaving precursor plasmid; intermediate en route to eDA110	This work
eDA89	7.9	pPICZα B-*GFP:FH*	DNA-parts prep. (GFP-FH fusion)	unpublished
eDA99	3.66	0.95-kb *LHR*^{Z} in pUC19 (*Bsa*I version)	DNA-parts prep.	This work
eDA101	3.66	0.95-kb *LHR*^{Z} in pUC19 (*BsmB*I version)	DNA-parts prep.	This work
eDA103	5.24	*Hyg*^{R}-*LHR*^{Z} part in pUC19 (*Bsa*I version) (with *AsiS*I in *Hyg*^{R})	DNA-parts prep.	This work
eDA105	4.83	*Zeo*^{R}-*LHR*^{Z} part in pUC19 (*BsmB*I version)	DNA-parts prep.	This work
eDA110	14.5	pUC-*PARS-A76-Cen3-LHR*^{A}- *Zeo*^{R}-*Kan*^{R}*(ΔI-SceI)-LHR*^{Z}	Intermediate plasmid en route to precursor plasmid *v*1	This work
eDA115	5.24	*Hyg*^{R}-*LHR*^{Z} in pUC19 (*Bsa*I version) (no *AsiS*I)	DNA-parts prep.	This work
eDA131	3.55	*telomere-I-SceI-telomere* in pUC19	DNA-parts prep. (Proto-telomeres*, Tel*)	This work
eDA137	14.9	pUC-*PARS-A76-CEN3-LHR*^{A}- *Zeo*^{R}-*Kan*^{R}*::(P*$_{AOX1}$*)I-SceI-LHR*^{Z} *AmpR::telomere-I-SceI-telomere*	Precursor plasmid *v*1	This work
eDA143	10.5	*PDI* in pPIC3.5K	Source of *PDI*	(Kerr, Herbert et al. 2021)
eDA144	4.6	1.9-kb *PARS-B413-ncDNA* part in pUC19	DNA-parts prep. (p-arm extension)	This work
eDA146	16.8	pUC-*PARS-A76-CEN3-PARS-B413-ncDNA-LHR*^{A}- *Zeo*^{R}-*Kan*^{R}*(ΔI-SceI)-LHR*^{Z}	Intermediate plasmid, en route to eDA155	This work
eDA155	17.3	pUC-*PARS-A76-CEN3-PARS-B413-ncDNA-LHR*^{A}- *Zeo*^{R}- *Kan*^{R}*::(P*$_{AOX1}$*)I-SceI-LHR*^{Z}*-AmpR::telomere-I-SceI-telomere*	Precursor plasmid *v*2	This work
eDA189	4.5	pUC-(*PARS-A76*)-*(P*$_{TEF1}$*)GFP(T*$_{CYC1}$*)*	DNA-parts prep. (*GFP* cassette)	This work

eDA191	4.5	pUC-(*PARS-A76)-(P_{TEF1})mCH(T_{CYC1})*	DNA-parts prep. (*mCH* cassette)	This work
eDA197	7.14	pUC-*LHR*^{E}-(P_{TEF1})*GFP*(T_{CYC1})-*Zeo*^{R}-*LHR*^{Z}	Assembly/repository of integration array	This work
eDA199	7.5	pUC-*LHR*^{E}-(P_{TEF1})*mCH*(T_{CYC1})-*Hyg*^{R}-*LHR*^{Z}	Assembly/repository of insertion array	This work
eDA201	15.8	pUC-*PARS-A76-CEN3-PARS-B413-ncDNA-LHR*^{E}-*GFP-Zeo*^{R}-*LHR*^{Z}-*AmpR::telomere-I-SceI-telomere*	Precursor plasmid *v*2b	This work
eDA226	4.8	pPICZ A-(P_{AOX1})*PDI*$_{H}$(T_{AOX1})	DNA-parts prep. (His-tagged *PDI*)	This work
eDA227	10.0	pUC-*LHR*^{A}-(P_{AOX1})*PDI*(T_{AOX1})-*LHR*^{E}-*HygR-LHR*^{Z}	Assembly/repository of insertion array	This work
eDA229	18.7	pUC-*PARS-A76-CEN3-PARS-B413-ncDNA-LHR*^{A}-(P_{AOX1})*PDI*(T_{AOX1})-*LHR*^{E}-*HygR-LHR*^{Z} -*AmpR::telomere-I-SceI-telomere*	Precursor plasmid *v*2a	This work
eDA250	12.8	pUC-*LHR*^{E}-(P_{AOX1})*GFP:FH*(T_{AOX1})-*LHR*^{D}-*ZeoR-LHR*^{Z}	Assembly/repository of integration array	This work

Table 3 *List of K. phaffii strains referred to in the current work*

K. phaffii strain	Genotype	Description (notes)	Landing Zone	Source
Background	CBS7435 (note: Mut^S)	Wild type (WT)	n/a	TFS
yDA34	CBS7435;eDA53	WT, transformed with framework plasmid	n/a	This work
yDA39	CBS743;eDA40	WT, transformed with episomal Zeo^R-carrying plasmid, as negative control for chromosome-loss assays	n/a	This work
yDA122	CBS743;linear eDA137	WT, transformed with linearised precursor plasmid *v*1 – [isolate no.1]	LHR^A- Zeo^R-Kan^R(Δ*I-SceI*)-LHR^Z	This work
yDA140	CBS7435;eDA37	WT, transformed with episomal, Hyg^R-containing plasmid as negative control for chromosome-loss assays	n/a	This work
yDA149	CBS7435;linear eDA137	WT, transformed with linearised precursor plasmid *v*1 – [isolate no.2]	LHR^A- Zeo^R-Kan^R(Δ*I-SceI*)-LHR^Z	This work
yDA174	CBS7435;linear eDA155	WT, transformed with linearised precursor plasmid *v*2 – [isolate no.1]	LHR^A- Zeo^R-Kan^R(Δ*I-SceI*)-LHR^Z	This work
yDA175	CBS7435;linear eDA155	WT, transformed with linearised precursor plasmid *v*2 – [isolate no.2]	LHR^A- Zeo^R-Kan^R(Δ*I-SceI*)-LHR^Z	This work
yDA177	CBS7435;linear eDA201	WT, transformed with precursor plasmid *v*2B – [isolate no.2]	LHR^E-P_{TEF}*GFP*-Zeo^R-LHR^Z	This work
yDA208	CBS7435 Δ*KU70*	WT with KU70-encoding gene knocked out (KO)	n/a	(Dalvie, Leal et al. 2020)
yDA218	CBS7435 Δ*KU70;*linear eDA201 (nChr 2B)	*KU70*-KO, transformed with linearised precursor *v*2B – [isolate no.1]	LHR^E-P_{TEF}*GFP*-Zeo^R-LHR^Z	This work
yDA221	CBS7435 P_{AOX1}::*mFH*(Zeo^R)	WT with genome-integrated murine mFH-expression cassette	n/a	This work
yDA226	CBS7435 Δ*KU70;*nChr 2B.1	*KU70*-KO, with nChr 2B.1 (after double-crossover HR performed on the landing zone of nChr 2B in yDA218)	LHR^E-(P_{TEF})*mCH*-Hyg^R-LHR^Z	This work
yDA232	CBS7435;linear eDA229	WT, transformed with linearised precursor plasmid *v*2A	LHR^A-(P_{AOX1})PDI_H(T_{AOX1})-	This work

	(nChr 2A)		*LHR^{E}-Hyg^{R}-LHR^{Z}*	
yDA245	CBS7435 P_{AOX1}::P_{AOX} *mFH(Zeo*^{R}*);* linear eDA229 (nChr 2A)	WT, with genome-integrated *mFH*-expression cassette, transformed with linearised precursor plasmid *v*2A	*LHR^{A}-(P_{AOX1})PDI_{H}(T_{AOX1})-LHR^{E}-Hyg^{R}-LHR^{Z}*	This work
yDA250	CBS7435 Δ*KU70* P_{AOX1}::P_{AOX1}*mFH(Zeo*^{R})	*KU70*-KO, with genome-integrated *mFH*-expression cassette	n/a	This work
yDA253	CBS7435 Δ*KU70;* linear eDA229 (nChr 2A)	*KU70*-KO, transformed with linearised precursor plasmid *v*2A	*LHR^{A}-(P_{AOX1})PDI_{H}(T_{AOX1})-LHR^{E}-Hyg^{R}-LHR^{Z}*	This work
yDA260	CBS7435 Δ*KU70* P_{AOX1}*::mFH(Zeo*^{R}*);* linear eDA229 (nChr 2A)	*KU70*-KO, with genome-integrated *mFH*-expression cassette, transformed with linearised precursor plasmid *v*2A	*LHR^{A}-(P_{AOX1})PDI_{H}(T_{AOX1})-LHR^{E}-Hyg^{R}-LHR^{Z}*	This work
yDA263	CBS7435 Δ*KU70* with nChr 2A.1	*KU70*-KO, with nChr 2A.1 (after double-crossover HR attempted on the landing zone of nChr 2A in yDA253)	*LHR^{A}-(P_{AOX1})PDI_{H}(T_{AOX1})-LHR^{E}-(P_{TEF})GFP(T_{TEF})-Zeo^{R}-LHR^{Z}*	This work
yDA264	CBS7435 Δ*KU70* P_{AOX1}*::mFH (Zeo*^{R}) P_{AOX1}*::PDIHIS (Hyg*^{R})	*KU70*-KO with genome-integrated *mFH* and *PDI_{H}* expression cassettes	n/a	This work
yDA275	CBS7435 Δ*KU70* nChr 2A.2	*KU70*-KO with nChr 2A.2 [clone 1] (after double-crossover HR attempted on the landing zone of nChr 2A in yDA253)	*LHR^{A}-(P_{AOX1})PDI_{H}(T_{AOX1})-LHR^{E}-(P_{AOX1})GFP:FH(T_{AOX1})-LHR^{D}-Zeo^{R}-LHR^{Z}*	This work
yDA277	CBS7435 Δ*KU70* nChr 2A.2	*KU70*-KO with nChr 2A.2 [clone 2] (after double-crossover HR attempted on the landing zone of nChr 2A in yDA253)	*LHR^{A}-(P_{AOX1})PDI_{H}(T_{AOX1})-LHR^{E}-(P_{AOX1})GFP:FH-LHR^{D}-Zeo^{R}*	This work
yDA286	CBS7435 Δ*KU70 nChr 2*	*KU70*-KO with nChr 2	*LHR^{A}- Zeo^{R}-Kan^{R}*(Δ*I-SceI*)-*LHR^{Z}*	This work

Table 4 *List of insertion and integration arrays*

Array	Int/ Ins*	Assembled from parts (size in kb)	Prepared from (oligos used for PCR)	First ligation product (size in kb)	Array size (kb)
LHR^{A}-(Kan^{R})-I-SceI-Zeo^{R}-LHR^{Z}	Int	LHR^{A} (0.85)	eDA8 (264/273)	n/a	~6.2
		Kan^{R} (0.98)	pGS-Man-II (GlycoSwitch)		
		I-SceI (2.2)	eDA27 (267/268)		
		Zeo^{R}-LHR^{Z} (2.12)	pGS-GnT-I (GlycoSwitch)		
LHR^{E}-GFP-Zeo^{R}-LHR^{Z}	Int	LHR^{E} (0.87)	eDA9 (335/336)	LHR^{E}-$(P_{TEF1})GFP$ (T_{CYC1}) (2.31)	~4.4
		$(P_{TEF1})GFP(T_{CYC1})$ (1.44)	eDA189 (302a/132a)		
		Zeo^{R}-LHR^{Z} (2.12)	eDA105 (308/318)		
LHR^{E}-mCH-Hyg^{R}-LHR^{Z}	Int/ Ins	LHR^{E} (0.87)	eDA9 (335/336)	LHR^{E}-$(P_{TEF1})mCH$ (T_{CYC1}) (2.28)	~4.8
		$(P_{TEF1})mCH(T_{CYC1})$ (1.41)	eDA191 (302a/132a)		
		Hyg^{R}-LHR^{Z} (2.54)	eDA115 (306/317)		
LHR^{A}-PDI^{H}-LHR^{E}-Hyg^{R}-LHR^{Z}	Int	LHR^{A} (0.85)	eDA8 (300/301)	LHR^{A}-$(P_{AOX1})PDI_{HIS}$ (T_{AOX1}) (3.7)	~7.11
		$(P_{AOX1})PDI_{HIS}$ (T_{AOX1}) (2.85)	eDA227 (302/303)		
		LHR^{E} (0.87)	eDA9 (335/336)	LHR^{E}-Hyg^{R}-LHR^{Z} (3.41)	
		Hyg^{R}-LHR^{Z} (2.54)	eDA115 (306/317)		
LHR^{E}-GFP:FH-LHR^{D} -Zeo^{R}-LHR^{Z}	Ins	LHR^{E} (0.9)	eDA9 (335/338)	LHR^{E}-$(P_{AOX1})GFP:FH$ (T_{AOX1}) (6.8)	~10.1
		$(P_{AOX1})GFP:FH(T_{AOX1})$ (5.9)	eDA89 (302/303)		
		LHR^{D} (1.1)	eDA9 (304/305)	LHR^{D}-Zeo^{R}-LHR^{Z} (3.2)	
		Zeo^{R}-LHR^{Z} (2.1)	eDA105 (308/318)		

Table 5 *Results of nanochromosome-loss assays performed for various strains*

**Strain ID**	Background strain (host)	Transformation with linearised versions of (and hence the **nanochromosome** that is potentially introduced)	% of Zeo- or Hyg-resistant isolates lost (red font) after growth in non-selective media*
yDA122	GS115	eDA137 (**nChr 1**)	10% loss 90% retain
yDA140	CBS7435	eDA37 (episomal plasmid)	88% loss 12% retain
yDA174	CBS7435	eDA155 (**nChr 2**)	17% loss 83% retain
yDA175	CBS7435	eDA155 (**nChr 2**)	16% loss 84% retain
yDA177	CBS7435	eDA201 (**nChr 2B**)	20% loss 80% retain
yDA218	CBS7435 *ΔKU70*	eDA201 (**nChr 2B**)	12% loss 88% retain
yDA226	CBS7435 *ΔKU70*	eDA201 (**nChr 2B.1**)	13% loss 87% retain
yDA232	CBS7435	eDA229 (**nChr 2A**)	17% loss 83% retain
yDA250	CBS7435 *ΔKU70* + genome-integrated mFH	none	98% loss 2% retain
yDA253	CBS7435 *ΔKU70*	eDA229 (**nChr 2A**)	19% loss 81% retain
yDA260	CBS7435 Δ*KU70* + genome-integrated mFH	eDA229 (**nChr 2A**)	19% loss 81% retain
yDA263	CBS7435 *ΔKU70* transformed w. linearised precursor plasmid *v*2A	eDA229 *i.e.* nChr 2 subsequently engineered to create **nChr 2A.1**	12% loss 88% retain
yDA264	CBS7435 *ΔKU70* with genome-integrated *Hyg*^{R})	none	16% loss 84% retain
yDA275	CBS7435 *ΔKU70 transformed w. linearised precursor plasmid* v2*A*)	eDA229 *i.e.* nChr 2 subsequently engineered to create **nChr2 A.2**	12% loss 88% retain
yDA277	(CBS7435 *ΔKU70 transformed with linearised precursor* v2*A*)	eDA229 *i.e.* nChr 2 subsequently engineered to create **nChr2 A.2**	11% loss 89% retain

* Transformed cells were cultured for at least ten generations in the absence of any selection, then an assay based on counting colonies on agar plates was performed to assess retention of antibiotic-resistance. The values listed here were calculated from the

total number of colonies counted growing on antibiotic-resistance selection medium, divided by the number of colonies counted growing on antibiotic-resistance *non*-selection medium. At least three biological replicates were performed for each strain. Standard deviations are illustrated by bars.

Table 6 A *Validation of primers sets for qPCR assays*. Slope and efficiency (%) values calculated from the sample dilution using StepOnePlus software (Thermo Fisher)

Gene	Target locus (amplicon length, bp)	Oligonucleotide primer pair	Slope	Efficiency range (%)
ACT1	Gene reference (137 bp)	35(F): TTCGTCGGTGACGAGGCTC<br>36(R): GGGGCCAGACGCAACTCG	-3.299	100.9
PDHPD A	Gene reference (132 bp)	258(F): GCGAAGCCGTTCTTCTCGAAG<br>259(R): CTGAACTCGCTCCTCCCCC	-3.350	98.84
rPDI[1]	Chaperon gene (135 bp)	24(F): GCCAGAGGACGCTTCTAACTTGG<br>25(R): GCTGGAGTGGAGTCGGAAGTG	-3.319	100.1
HPH	Hygromycin resistance gene (135 bp)	41(F): CGACAGCGTCTCCGACCTG<br>42(R): GGCGCAGCTATTTACCCGC	-3.245	103.3
mCFH[1]	Murine CFH (179 bp)	212(F): CCGTCGCCATTCTCGACTC<br>215(R): GCAGCCGAAACCGATCAGGAG	-3.32	100.1
CEN3 core	Centromere (129 bp)	364(F): CACGCGAGATGCACTCAGTGC<br>365(R): CGTAGCTGCTTCATCCGGC	-3.213	104.7
LHR[C]	synthetic DNA (120 bp)	370(F): CGTTACACGGCAGTCCTGTTCC<br>371(R): CTTGTAGCTCAGCTTACCCACC	-3.223	104.3

[1] – gene codon optimized

Table 6 B *Estimated GCN (normalised to GCN for ACT1 = 1)*

		**Analysed strains (all on background of CBS7435 *ΔKU70*)**						
**GENE**	**Copy no. /Ave. C_T**	yDA218 nChr 2B	yDA226 nChr 2B	yDA253 nChr 2A	yDA263 nChr 2A	yDA277 nChr 2A	yDA260 P_{AOX1}::*mFH*; nChr 2A	yDA264 P_{AOX1}::*mFH*-P_{AOX1}::PDI^H
ACT1 (Ref)	**Copy no.**	**1**	**1**	**1**	**1**	**1**	**1**	**1**
	Average C_T	21.15±0.03	21.63±0.02	22.57±0.04	19.08±0.02	20.46±0.04	18.82±0.08	16.26±0.3
PDI^H	**Copy no.**	n/a	n/a	1.1	1.0	1.2	1.1	1.2
	Average C_T	n/a	n/a	22.37±0.19	19.28±0.01	20.21±0.1	18.65±0.23	15.98±0.03
HPH	**Copy no.**	n/a	**1.1**	**0.8**	**0.2**	**0.2**	**0.8**	**0.9**
	Average C_T	n/a	21.52±0.12	22.88±0.26	21.17±0.22	22.75±0.13	19.14±0.25	16.44±0.13
CEN3	**Copy no.**	**2.1**	**2.2**	**2.1**	**2.1**	**2.3**	**2.3**	**1.8**
	Average C_T	20.06±0.02	20.50±0.03	22.53±0.13	18.01±0.01	19.25±0.11	17.60±0.08	15.43±0.14
LHR^C	**Copy no.**	**0.9**	**1.0**	**0.9**	**0.9**	**0.8**	**1.0**	**0.1**
	Average C_T	21.25±0.07	21.64±0.06	22.67±0.03	19.28±0.01	20.8±0.05	18.81±0.22	19.95±0.03
mFH	**Copy no.**	n/a	n/a	n/a	n/a	n/a	**1.1**	**1.2**
	Average C_T	n/a	n/a	n/a	n/a	n/a	18.66±0.22	15.95±0.08
PDHPDA (2nd Ref)	**Copy no.**	**0.9**	**0.9**	**0.9**	**1.1**	**1.0**	**1.0**	**0.9**
	Average C_T	21.31±0.21	21.73±0.09	22.67±0.01	18.91±0.05	20.49±0.07	18.79±0.14	16.45±0.2

Average C_T (cycle threshold) values were assayed from two biological sample repeats in three technical replicates. GCNs were calculated by normalizing C_T values separately with respect to the housekeeping gene *ACT1*. *PDHPDA* is an additional control; both *ACT1* and PDHPDA are assumed to be present as single copies. The *HPH* gene encodes hygromycin B phosphotransferase.

REFERENCES

Dalvie, N. C., J. Leal, C. A. Whittaker, Y. Yang, J. R. Brady, K. R. Love and J. C. Love (2020). "Host-Informed Expression of CRISPR Guide RNA for Genomic Engineering in Komagataella phaffii." ACS Synth Biol **9**(1): 26-35.

Kerr, H., A. P. Herbert, E. Makou, D. Abramczyk, T. H. Malik, H. Lomax-Browne, Y. Yang, I. Y. Pappworth, H. Denton, A. Richards, K. J. Marchbank, M. C. Pickering and P. N. Barlow (2021). "Murine Factor H Co-Produced in Yeast With Protein Disulfide Isomerase Ameliorated C3 Dysregulation in Factor H-Deficient Mice." Front Immunol **12**: 681098.

Shen, Y., Y. Wang, T. Chen, F. Gao, J. Gong, D. Abramczyk, R. Walker, H. Zhao, S. Chen, W. Liu, Y. Luo, C. A. Müller, A. Paul-Dubois-Taine, B. Alver, G. Stracquadanio, L. A. Mitchell, Z. Luo, Y. Fan, B. Zhou, B. Wen, F. Tan, J. Zi, Z. Xie, B. Li, K. Yang, S. M. Richardson, H. Jiang, C. E. French, C. A. Nieduszynski, R. Koszul, A. L. Marston, Y. Yuan, J. Wang, J. S. Bader, J. Dai, J. D. Boeke, X. Xu, Y. Cai and H. Yang (2017). "Deep functional analysis of synII, a 770-kilobase synthetic yeast chromosome." Science **355**(6329).
